# Identification of shared biological features in four different lung cell lines infected with SARS-CoV-2 virus through RNA-seq analysis

**DOI:** 10.3389/fgene.2023.1235927

**Published:** 2023-08-16

**Authors:** Xiaoxi Zhang, Seungjun Ahn, Peihua Qiu, Somnath Datta

**Affiliations:** ^1^ Department of Biostatistics, University of Florida, Gainesville, FL, United States; ^2^ Department of Population Health Science and Policy, Icahn School of Medicine at Mount Sinai, New York, NY, United States

**Keywords:** SARS-CoV-2, COVID-19, RNA-seq, lung cell lines, differential expression analysis, pathway analysis, differential network analysis

## Abstract

The COVID-19 pandemic caused by SARS-CoV-2 has resulted in millions of confirmed cases and deaths worldwide. Understanding the biological mechanisms of SARS-CoV-2 infection is crucial for the development of effective therapies. This study conducts differential expression (DE) analysis, pathway analysis, and differential network (DN) analysis on RNA-seq data of four lung cell lines, NHBE, A549, A549.ACE2, and Calu3, to identify their common and unique biological features in response to SARS-CoV-2 infection. DE analysis shows that cell line A549.ACE2 has the highest number of DE genes, while cell line NHBE has the lowest. Among the DE genes identified for the four cell lines, 12 genes are overlapped, associated with various health conditions. The most significant signaling pathways varied among the four cell lines. Only one pathway, “cytokine-cytokine receptor interaction”, is found to be significant among all four cell lines and is related to inflammation and immune response. The DN analysis reveals considerable variation in the differential connectivity of the most significant pathway shared among the four lung cell lines. These findings help to elucidate the mechanisms of SARS-CoV-2 infection and potential therapeutic targets.

## 1 Introduction

As of 30 April 2023, the coronavirus disease 2019 (COVID-19) pandemic caused by severe acute respiratory syndrome coronavirus 2 (SARS-CoV-2) has led to more than 765 million confirmed cases and nearly 7 million deaths worldwide, reported to the World Health Organization (WHO). It’s reported that SARS-CoV-2 infection is similar to other respiratory infections such as SARS-CoV and MERS-CoV, which caused outbreaks in Southern China in 2003 and Saudi Arabia in 2012, respectively (Prompetchara et al., 2020). This similarity is attributed to the fact that all three viruses belong to the coronavirus family (Pustake et al., 2022). Genomic sequencing has shown that SARS-CoV-2 shares approximately 79% similarities with SARS-CoV and 50% similarities with MERS-CoV. Recent research indicates that the identifications of molecular mechanisms (Zhang et al., 2021a), cellular functions (Alipoor et al., 2021), and biological network activities (Habibi et al., 2021; Guo et al., 2022) are important to better understand the intricacies of disease biology and potentially lead to new therapies in treating COVID-19. The virus can invade cellular processes by moving proteins and genetic material from cells, resulting in the formation of new virus particles (Cohen, 2016). With regards to the SARS-CoV-2 virus, it has a detrimental effect on both lung epithelial cells (John et al., 2021) and lung adenocarcinoma cell lines (Stewart et al., 2021).

Our research of interest lies in comparing these four distinct lung cell lines: normal human bronchial epithelial cell line (NHBE), A549 lung adenocarcinoma cell line (A549), A549 expressing angiotensin-converting enzyme 2 receptors (A549.ACE2), and Calu-3 lung epithelial cell line (Calu3). This comparison is driven by the RNA-seq data (Blanco-Melo et al., 2020), providing information solely for these four specific lung cell lines. Rayner et al. (2019) state that NHBE, which is the primary lung epithelial cell, is widely employed as *in vitro* lung model for assessing the fundamental events that contribute to respiratory diseases. Blanco-Melo et al. (2020) and Vishnubalaji et al. (2020) indicate that A549 has a relatively low susceptibility to SAR-CoV-2 infection. Nevertheless, if A549 expresses angiotensin-converting enzyme 2 (ACE2) receptors, it can become compatible with SARS-CoV-2, as the exogenous expression of ACE2 allows SARS-CoV-2 to replicate in A549 cell (Blanco-Melo et al., 2020). In our study, we utilize A549.ACE2 to denote the A549 that has been modified to express ACE2 receptors. Calu3 is suitable for investigating innate immune responses to SARS-CoV-2 infection, as it reproduces IFN induction when exposed to SARS-CoV-2 (Rebendenne et al., 2021). These cells are of great significance in the study of SARS-CoV-2 infections and in the exploration of medical strategies through real-life experiments.

Recent studies related to COVID-19 disease have shed light on various aspects of the disease, including its pathogenesis, mechanisms of action, and potential therapeutic interventions. Here are some key findings. Draghici et al. (2021) examined the mechanisms of SARS-CoV-2 infection in NHBE, A549, and COVID-19 lung tissues. The analysis identified methylprednisolone as a promising therapy, which was confirmed by the survival analysis of clinical data. Blanco-Melo et al. (2020) conducted a comparison of the transcriptional response to the SARS-CoV-2 virus with other respiratory viruses such as influenza A virus (IAV) and respiratory syncytial virus (RSV). They concluded that the hallmark features of COVID-19 disease are reduced innate antiviral defenses and exuberant inflammatory cytokine production. Wu et al. (2021) conducted pathway enrichment analysis using two types of lung epithelial cells infected with SARS-CoV-2: A549 cells with overexpression of ACE2 and NHBE cells. They found that the main cause of the inability to eliminate SARS-CoV-2 is immune dysregulation and interferon malfunction. Chen et al. (2021) identified common mechanisms between SARS-CoV-2 infection and human cancers by conducting differential expression (DE) analysis and pathway analysis on three different lung cell lines (NHBE, A549, and Calu3) and performing a meta-analysis across various datasets. Daamen et al. (2021) investigated the pathogenesis of COVID-19 in COVID-19 patients by analyzing the lung, blood, and airway responses and discovered the dynamic nature of the inflammatory response to SARS-CoV-2. They also proposed various potential therapeutic suggestions based on their findings. A549 cells, lacking ACE2 expression, could still be infected by SARS-CoV-2, although its replication of SARS-CoV-2 is significantly higher in A549.ACE2 cells (Yang et al., 2020).

Although many studies have investigated COVID-19 using RNA-seq data from different cell lines, few have examined the common and unique biological characteristics among NHBE, A549, A549.ACE2, and Calu3 cell lines based on DE analysis, pathway analysis, and differential network (DN) analysis. Additionally, most of these studies have used only one approach for DE analysis, which could lead to biased results. In this study, we conduct a comparative analysis of the four different lung cell lines, namely, NHBE, A549, A549.ACE2, and Calu3. Our approaches involve DE analysis, pathway analysis, and DN analysis to identify both unique and common disease mechanisms among these cell lines. The structure of this paper is as follows. In Section 2, we provide a detailed description of the dataset used in our analysis, as well as our analysis plan and suggested statistical models. The results of the DE analysis, pathway analysis, and DN analysis are presented in Section 3. Finally, in Section 4, we provide some concluding remarks.

Our research project falls within the scope of the Disease Maps to Modeling COVID-19 research topic at the Critical Assessment of Massive Data Analysis (CAMDA) Annual Conference 2022.

## 2 Materials and methods

### 2.1 RNA-seq data

The RNA-seq data was obtained from the Gene Expression Omnibus (GEO) under the accession number GSE147507 (Blanco-Melo et al., 2020). To be specific, we incorporated Series1, Series2, Series5, Series6, Series7, and Series16 from the dataset. The dataset consists of 21797 genes and includes independent biological triplicates of four different human lung cell lines: primary human lung epithelium cell line (NHBE), transformed lung alveolar cell line (A549), transformed lung alveolar transduced with a vector expressing human ACE2 (A549.ACE2), and transformed lung-derived Calu-3 cells (Calu3), with mock treatment and SARS-CoV-2 infection as our research interest. Genes with low expression are filtered out based on a cutoff of median log_2_ transformed counts per gene per million mapped reads (CPM). After removing genes with a median log_2_(CPM) below −1, only 13111 genes are kept for the subsequent analysis. The right panel in Supplementary Figure S1 shows the normalized histogram of median log_2_(CPM) after removing the lowly expressed genes, which is in contrast to the highly left-skewed histogram before the filtering process, that is, shown in the left panel of Supplementary Figure S1.

### 2.2 Analysis plan

The study is designed to compare the mock-treated group and SARS-CoV-2-infected group in four different lung cell lines (NHBE, A549, A549.ACE2, and Calu3) with the aim of identifying singular and common DE genes, influenced pathways, and DN in these cell lines infected with the SARS-CoV-2 virus.

For A549.ACE2, the independent biological triplicates are also mock-treated or SARS-CoV-2-infected with or without Ruxolitinib (Rux) pre-treatment. Rux is verified as an effective and safe treatment for COVID-19 patients (La Rosée et al., 2020). In our analysis, we treat the independent biological triplicates infected with SARS-CoV-2 with and without Rux pre-treatment as one group based on the following two reasons: 1) The multidimensional scaling (MDS) and heatmap are techniques for visualizing the distances between two groups (cf., Mead, 1992; Wilkinson and Friendly, 2009). Both the MDS plot and the heatmap show a tiny difference between the independent biological triplicates infected with SARS-CoV-2 with and without Rux pre-treatment (cf., Supplementary Figures S2, S3). 2) Comparing DE genes between the mock-treated group and SARS-CoV-2-infected with Rux group and DE genes between the mock-treated group and SARS-CoV-2-infected without Rux group, there are negligible differences between these two lists of DE genes (cf., Supplementary Tables S1, S2).

A549.ACE2 is a modified version of A549 cells that expresses ACE2, the receptor for SARS-CoV-2 viral entry (Samavati and Uhal, 2020). In our study, we treat the independent biological triplicates of A549.ACE2 and A549 as distinct groups. We observe that similar to the analysis of Rux treatment, the difference between the independent biological triplicates of A549.ACE2 and A549 is smaller than other pairs of cell lines based on the MDS plot and heatmap. This is reasonable given that A549.ACE2 is a variant of A549. However, there are a small number of overlapping DE genes between the mock-treated and SARS-CoV-2-infected groups in both A549.ACE2 and A549 (cf., Supplementary Figures S2, S3; Supplementary Tables S3, S4).

### 2.3 Statistical methods

The objective of this study is to identify the singular and common DE genes, influenced pathways, and DN in the four lung cell lines (NHBE, A549, A549.ACE2, and Calu3) infected with the SARS-CoV-2 virus. All DE analysis, Pathway analysis, and DN analysis are performed based on the four pairs of comparison between the mock-treated group and SARS-CoV-2-infected group in each of the four cell lines (NHBEMock vs. NHBESARS.CoV.2, A549Mock vs. A549SARS.CoV.2, A549.ACE2Mock vs. A549.ACE2SARS.CoV.2, and Calu3Mock vs. Calu3SARS.CoV.2). Below, we outline the statistical methods used for the analysis of DE genes, pathways, and DN. All statistical analyses are conducted using R version 4.0.2 (R Foundation for Statistical Computing, Vienna, Austria).

#### 2.3.1 Differential expression analysis

DE genes are defined as genes that exhibit a difference in expression between groups. In the fields of clinical trials and drug development, DE genes play an important role in the understanding of underlying disease mechanisms, the identification of potential biomarkers, the discovery of therapeutic targets, and the generation of gene signatures for diagnostic purposes. In our study, the DE analysis is conducted to find the DE genes in the four lung cell lines (NHBE, A549, A549.ACE2, and Calu3) infected with the SARS-CoV-2 virus. To prevent bias and validate the results of DE genes, we use multiple methods rather than relying on a single approach. Three commonly used R packages for DE analysis are utilized in our analysis, which are *DESeq2* (Love et al., 2014), *edgeR* (Robinson et al., 2010), and *limma* (Ritchie et al., 2015), respectively. While these three methods have similar goals, there are some key differences in the normalization methods and statistical models that they use.

Three different normalization methods are utilized in these three DE methods (Dillies et al., 2013). The first method, employed by *DESeq2*, is called size factor normalization, which involves dividing the counts for each gene by a size factor that accounts for differences in library size. The second method, used by *edgeR*, is called trimmed mean of M-values (TMM), which adjusts for differences in library size and composition by scaling the counts for each sample to ensure that the average log-fold change between samples is zero. Finally, *limma* employs a method called quantile normalization, which adjusts for differences in library size and composition by matching the distribution of expression values across samples. As for statistical modeling techniques employed in this study, *DESeq2* utilizes the negative binomial (a.k.a. gamma-poisson) distribution. Similarly, *edgeR* employs negative binomial-based models, with the addition of an empirical Bayes procedure to shrink the dispersions. *Limma*, on the other hand, utilizes a linear modeling-based method and incorporates an empirical Bayes approach to borrow information between genes. To adjust *p*-values for multiple comparisons, the Benjamini–Hochberg correction (Benjamini and Hochberg, 1995) is applied to all three differential expression methods and the significance threshold is set at 0.05.

After obtaining the ordered lists of DE genes for each of the four cell lines (NHBE, A549, A549.ACE2, and Calu3) by different DE methods (i.e., *DESeq2*, *edgeR*, and *limma*), the R package *RankAggreg* (Pihur et al., 2009) can be employed to aggregate these ordered lists based on the ranks using the Genetic Algorithm (Golberg, 1989).

#### 2.3.2 Pathway analysis

DE analysis has a limitation that it can lead to the identification of a large number of DE genes between sample groups, making it challenging to apply visualization techniques and interpret in the biological context about these genes. To extract maximum information from RNA-seq data and gain a better understanding of the biological context of these DE genes and their potential role in disease or other biological processes, researchers can identify the signaling pathways affected by the observed changes. Signaling pathways consist of a group of genes that provides information about the fundamental cellular mechanisms and interactions required for the development of morphology and organs (Sanz-Ezquerro et al., 2017). In our study, the R package *SPIA* (Tarca et al., 2009) is utilized to examine the pathway topology, which includes listing the component genes and illustrating their interactions within the pathway (García-Campos et al., 2015). *SPIA* considers two pieces of evidence to evaluate the impact of DE genes on signaling pathways. Firstly, a “classical” pathway enrichment analysis is used to assess the number of DE genes observed in a given pathway. Secondly, the actual perturbation (activation or inhibition) of a given pathway under a specific condition is measured. Here, perturbation denotes the disturbance that induces changes in the gene-gene association. Human signaling pathways from the Kyoto Encyclopedia of Genes and Genomes (KEGG) database (Ogata et al., 1999), which is a knowledge open-source database for biological pathways, are used for this analysis. It should be mentioned that we consider pathways with an adjusted *p*-value below 0.05 to be statistically significant.

#### 2.3.3 Differential network analysis

DN analysis is a method to identify group-specific changes in measures of differential connectivity (DC) by comparing networks between different sample groups (Gill et al., 2010; Grimes et al., 2019). In the analysis of RNA-seq data, the RNA-seq co-expression network is a type of differential network (Ballouz et al., 2015; Grimes et al., 2019), which comprise nodes representing genes and edges representing gene-gene associations (Mitra et al., 2013). The changes in the topology between two networks may suggest variations in cellular activity (Lu et al., 2007). In our study, we utilize the R package *dnapath* (Grimes et al., 2019) to perform DN analysis that integrates gene regulatory pathways into a DN analysis. The *dnapath* package can identify the DC pathways, DC genes, and DC edges by computing DC scores, with *p*-values calculated via monotonized permutation tests. The DN analysis is performed on pathways with sizes ranging from 10 to 200 in our study. The pathway information used in our analysis is obtained from the Reactome database (Jassal et al., 2020), which is a publicly available resource of biological pathways. The *dnapath* package is applied to the RNA-seq data from the literature (Ahn et al., 2021) to investigate the influence of tumor purity (TP) on gene expression data.

## 3 Results

### 3.1 Differential expression analysis

Table 1 displays the results of the three distinct DE methods, *DESeq2*, *edgeR*, and *limma*, for identifying DE genes among a total of 13111 genes in the four lung cell lines (NHBE, A549, A549.ACE2, and Calu3). The percentage of DE genes for each cell line is presented in the parenthesis in each entry of the table. It is worth noting that these methods exhibit minor differences, except for the *limma* method, which can detect almost 1.5 times as many DE genes for A549 in comparison with *DESeq2* or *edgeR*. Among the four cell lines, NHBE has the lowest percentage of DE genes, which is approximately 0.2%, whereas A549.ACE2 has the highest percentage of DE genes, around 60%, which is consistent with the fact that ACE2 serves as the receptor for SARS-CoV-2 viral entry (Samavati and Uhal, 2020).

**TABLE 1:** Numbers of detected DE genes in the four lung cell lines (NHBE, A549, A549.ACE2, and Calu3) using the three DE methods (*DESeq2*, *edgeR*, and *limma*). The numbers in parentheses denote the percentages of detected DE genes for each cell line among a total of 13111 genes.

Cell lines	Methods		
	*DESeq2*	*edgeR*	*limma*
NHBE	34 (0.3%)	27 (0.2%)	25 (0.2%)
A549	1794 (13.7%)	1561 (11.9%)	2605 (19.9%)
A549.ACE2	7926 (60.5%)	7728 (58.9%)	7614 (58.1%)
Calu3	4266 (32.5%)	4082 (31.1%)	4481 (34.2%)

Given such a large number of DE genes, placing emphasis on the most biologically relevant genes can lead to more valuable insights in practical applications such as discovering biomarkers and potential therapeutic targets. Therefore, a list of the top 10 DE genes for each cell line using the three different DE methods is generated and presented in Table 2. It can be seen from the table that there is a little variation in the top 10 DE genes identified for each cell line by different DE methods. *RankAggreg* is then used to combine the ranked lists of DE genes from each cell line, based on the ranks obtained from the three DE methods via the Genetic Algorithm (Table 2).

**TABLE 2:** Top ten DE genes in the four lung cell lines (NHBE, A549, A549.ACE2, and Calu3) using three DE methods (*DESeq2*, *edgeR*, and *limma*), as well as the Rank Aggregation Method *RankAggreg*.

Cell lines	Methods			
	*DESeq2*	*edgeR*	*limma*	*RankAggreg*
NHBE	CXCL5, PLAT	CSF3, CXCL5	TNFAIP2, SAA2	CSF3, CXCL5
	CSF3, TNFAIP2	TNFAIP2, PLAT	IFITM10, SAA1	TNFAIP2, PLAT
	ZC3H12A, IFITM10	IFITM10, ZC3H12A	ZC3H12A, CSF3	ZC3H12A, IFITM10
	BPGM, OLFML2A	OLFML2A, BPGM	MMP9, BPGM	OLFML2A, BPGM
	TNIP1, KYNU	TNIP1, C1QTNF1	PLAT, TNIP1	TNIP1, MMP9
A549	BTG3, CLDN1	BTG3, CLDN1	BTG3, ANXA3	BTG3, CLDN1
	TXNIP, ANXA3	SERPINB7vGAS5	SYNGR3, NT5DC2	ANXA3, GAS5
	GAS5, SERPINB7	LAMC2, ANXA3	CLDN1, OD2	LAMC2, SERPINB7
	LAMC2, ADM2	TXNIP, SAT1	IRF9, SYTL2	TXNIP, IRF9
	NEB, EREG	MTHFD2, EREG	NEB, MGAT5B	NEB, EREG
A549.ACE2	IER5, HIST2H2BE	IER5, HIST2H2BE	HIST2H2BE,IER5	IER5, HIST2H2BE
	EGR1, DUSP8	DUSP8, EGR1	DUSP8, PCF11	DUSP8, EGR1
	PCF11, NFKBIE	PCF11, NFKBIE	BCL3, EPM2AIP1	PCF11, NFKBIE
	BCL3, NFKBIA	BCL3, ZC3H4	ZC3H4, PPM1D	BCL3, ZC3H4
	ZC3H4, CCNL1	CCNL1, EGR2	CCNL1, NFKBIA	CCNL1, NFKBIA
Calu3	THBS1, DAPP1	THBS1, TNFAIP2	THBS1, TNFAIP2	THBS1, TNFAIP2
	TNFAIP2, ADRB2	DAPP1, NUAK2	PTGER4, MB21D2	DAPP1, NUAK2
	PLAT, MAP3K8	MAP3K8, PLAT	MAP3K8, B4GALT5	MAP3K8, PLAT
	NUAK2, B4GALT5	ADRB2, TNF	LGALS9, PLAT	ADRB2, B4GALT5
	TNF, IRS2	B4GALT5, IL12A	PTAFR, CTGF	TNF, PTAFR

**FIGURE 1:**
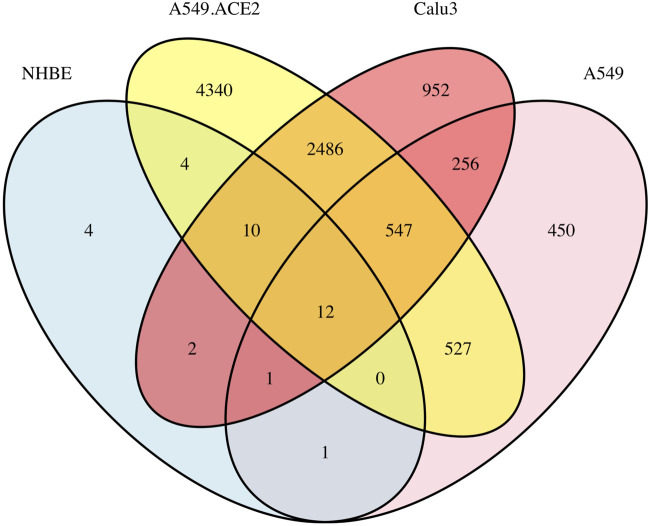
A Venn diagram illustrating the overlap of DE genes among the four lung cell lines (NHBE, A549, A549.ACE2, and Calu3) based on the results of *DESeq2*.

Based on the *DESeq2* results, we plot a Venn diagram (cr., Figure 1) to visualize the overlapping DE genes among the four lung cell lines, NHBE, A549, A549.ACE2, and Calu3. The analysis shows that there are 14 overlapping DE genes between A549 and NHBE, 26 overlapping DE genes between A549.ACE2 and NHBE, and 25 overlapping DE genes between Calu3 and NHBE. Moreover, there are 1086 overlapping DE genes between A549 and A549.ACE2, 816 overlapping DE genes between A549 and Calu3, and 3055 overlapping DE genes between A549.ACE2 and Calu3. Notably, 12 DE genes are shared among all four cell lines, including NHBE, A549, Calu3, and A549.ACE2. These genes are SAA2, BIRC3, TNFAIP2, IL32, CSF3, C1QTNF1, C3, KYNU, CXCL5, CXCL3, VNN1, and SOD2. These genes have been identified that are associated with both severe COVID-19 and various other health conditions. SAA2, for example, is linked to inflammation and tissue (e.g., cancer cell lines) injury (Malle et al., 2009) and has been identified as a biomarker of severe COVID-19 and poor prognosis (Li et al., 2020). BIRC3 is an upregulated gene, also verified in our analysis, in glioblastoma and leads to therapeutic resistance due to its critical role in the NF-*κ*B signaling pathway (Wang et al., 2016). It has also been identified as one of the six common hub proteins involved in SARS-CoV-2 infection and the risk factors related to COVID-19 (Nain et al., 2021). Additionally, CXCL3, a member of the CXC-type chemokine family, is known to be involved in the development and progression of various types of cancer (Lou et al., 2023) and has also been linked to COVID-19 (Chua et al., 2020).

For more comprehensive information about DE genes, including the number of upregulated and downregulated genes and the complete table regarding the results of the top 10 significant DE genes for each cell line type, along with the corresponding volcano plots, please refer to Supplementary Tables S5–S9 and Supplementary Figure S4.

### 3.2 Pathway analysis

**FIGURE 2:**
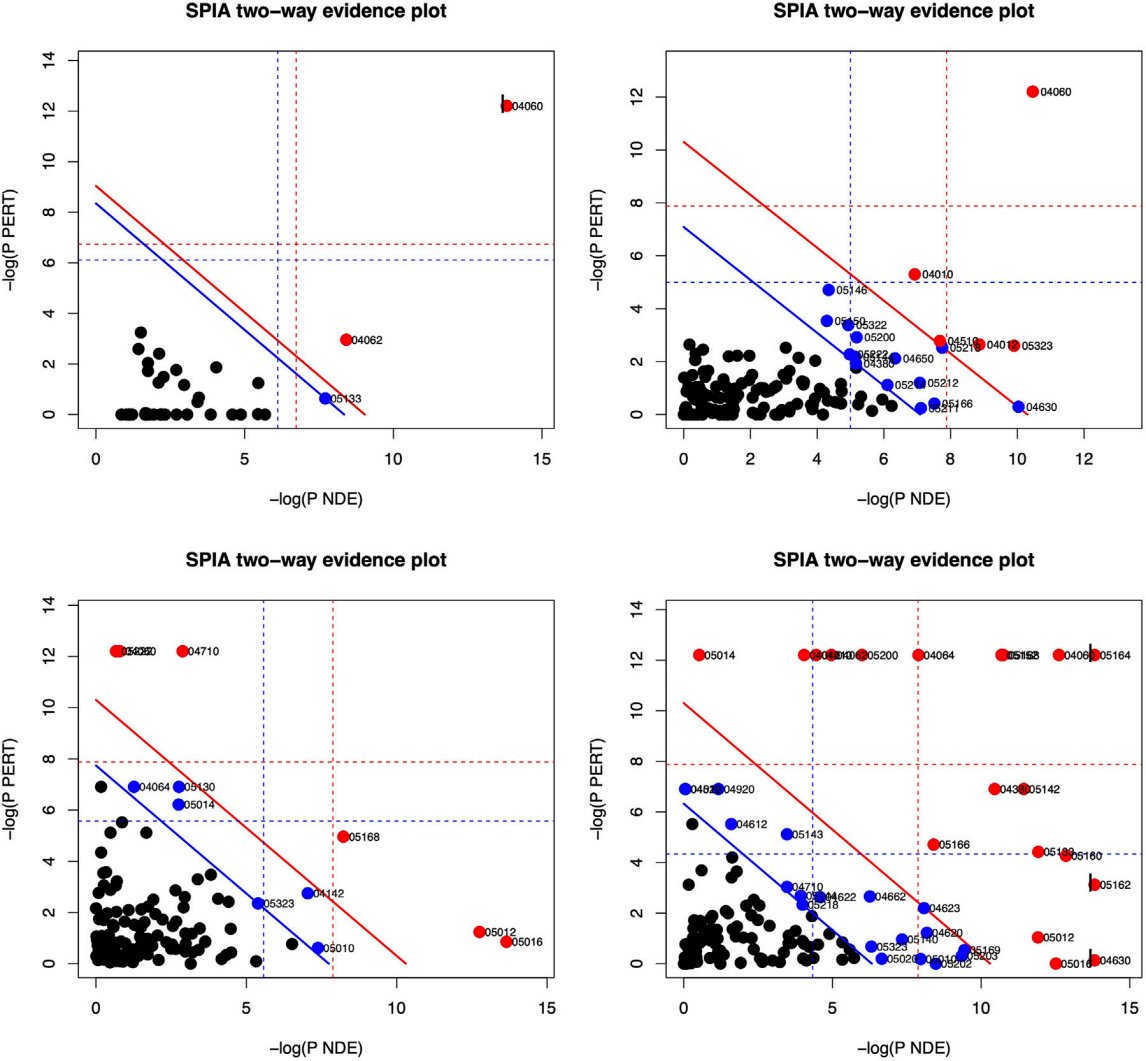
SPIA two-way evidence plots for the four lung cell lines: NHBE (top-left), A549 (top-right), A549.ACE (bottom-left), and Calu3 (bottom-right). The most significant pathways in the experiment are represented by the red dots. The significance is determined by measuring the log of pNDE and the log of pPERT.

Based on the DE genes identified by *DESeq2* for each lung cell line, *SPIA* is used to investigate the significant signaling pathways for each cell line. The top 10 significant signaling pathways for each lung cell line, as determined by *SPIA*, are presented in Tables 3–6. These pathways play crucial roles in the functioning of the four lung cell lines, highlighting their potential relevance in respiratory infections and diseases. Furthermore, Figure 2 displays the corresponding SPIA two-way evidence plots.

**TABLE 3:** Ten most significant pathways identified by *SPIA* for the lung cell line NHBE.

Name	pSize	NDE	pNDE	tA	pPERT	pG	pGFdr	Status
Cytokine-cytokine receptor interaction	117	6	0.00	−11.21	0.00	0.00	0.00	Activated
Chemokine signaling pathway	114	4	0.00	−12.63	0.05	0.00	0.00	Activated
Pertussis	57	3	0.00	1.99	0.53	0.00	0.03	Inhibited
Complement and coagulation cascades	37	2	0.00	−5.12	0.29	0.01	0.10	Activated
Apoptosis	77	2	0.02	4.68	0.16	0.02	0.16	Inhibited
Transcriptional misregulation in cancer	115	3	0.00	0.00	1.00	0.02	0.16	Inhibited
Tuberculosis	125	3	0.00	0.08	0.99	0.03	0.16	Inhibited
Legionellosis	50	2	0.01	0.00	1.00	0.05	0.22	Inhibited
Toxoplasmosis	94	1	0.02	2.52	0.04	0.05	0.22	Inhibited
Rheumatoid arthritis	58	2	0.01	0.00	1.00	0.06	0.22	Inhibited

The columns include the number of genes on the pathway (pSize), the number of differentially expressed (DE) genes on the pathway (NDE), the observed total perturbation accumulation in the pathway (tA), the probability of observing at least NDE, genes on the pathway using a hypergeometric model (pNDE), the probability of observing a total accumulation more extreme than tA only by chance (pPERT), the *p*-value obtained by combining pNDE, and pPERT (pG), the False Discovery Rate (pGFdr), and the direction in which the pathway is perturbed (activated or inhibited) (status). The columns in the table are arranged in the following order: Name, ID, pSize, NDE, pNDE, tA, pPERT, pG, pGFdr, and status. Pathways with pGFdr, below 0.05 are considered as significant.

**TABLE 4:** Ten most significant pathways identified by *SPIA* for the lung cell line A549.

Name	pSize	NDE	pNDE	tA	pPERT	pG	pGFdr	Status
Cytokine-cytokine receptor interaction	117	33	0.00	−20.00	0.00	0.00	0.00	Activated
Rheumatoid arthritis	58	20	0.00	−2.21	0.07	0.00	0.00	Activated
MAPK signaling pathway	207	45	0.00	−19.18	0.01	0.00	0.00	Activated
ErbB signaling pathway	76	23	0.00	−20.70	0.07	0.00	0.00	Activated
Focal adhesion	161	38	0.00	−19.11	0.06	0.00	0.01	Activated
Jak-STAT signaling pathway	85	26	0.00	−0.68	0.75	0.00	0.01	Activated
Melanoma	48	16	0.00	−15.98	0.08	0.00	0.01	Activated
Amoebiasis	76	18	0.01	5.57	0.01	0.00	0.02	Inhibited
Natural killer cell mediated cytotoxicity	79	21	0.00	−19.59	0.12	0.00	0.03	Activated
Systemic lupus erythematosus	18	7	0.01	−4.76	0.03	0.00	0.03	Activated

Pathways with pGFdr, below 0.05 are considered as significant.

**TABLE 5:** Ten most significant pathways identified by *SPIA* for the lung cell line A549.ACE2.

Name	pSize	NDE	pNDE	tA	pPERT	pG	pGFdr	Status
Circadian rhythm - mammal	20	16	0.06	26.04	0.00	0.00	0.00	Inhibited
Huntington’s disease	151	119	0.00	−2.89	0.43	0.00	0.00	Activated
Parkinson’s diseas	93	77	0.00	9.62	0.29	0.00	0.00	Inhibited
Herpes simplex infection	142	106	0.00	−39.95	0.01	0.00	0.00	Activated
Cytokine-cytokine receptor interaction	117	72	0.45	−70.01	0.00	0.00	0.00	Activated
Small cell lung cancer	77	47	0.51	41.66	0.00	0.00	0.00	Inhibited
Lysosome	112	84	0.00	1.34	0.06	0.00	0.01	Inhibited
Pathogenic *Escherichia coli* infection	47	34	0.06	35.66	0.00	0.00	0.01	Inhibited
Amyotrophic lateral sclerosis (ALS)	44	32	0.06	−39.56	0.00	0.00	0.02	Activated
NF-kappa B signaling pathway	68	44	0.28	−39.84	0.00	0.00	0.04	Activated

Pathways with pGFdr, below 0.05 are considered as significant.

**TABLE 6:** Ten most significant pathways identified by *SPIA* for the lung cell line Calu3.

Name	pSize	NDE	pNDE	tA	pPERT	pG	pGFdr	Status
Influenza A	125	70	0.00	−40.90	0.00	0.00	0.00	Activated
Cytokine-cytokine receptor interaction	117	62	0.00	−106.10	0.00	0.00	0.00	Activated
Herpes simplex infection	142	70	0.00	−71.60	0.00	0.00	0.00	Activated
Tuberculosis	125	63	0.00	−84.70	0.00	0.00	0.00	Activated
NF-kappa B signaling pathway	68	36	0.00	−59.10	0.00	0.00	0.00	Activated
Chagas disease (American trypanosomiasis)	78	44	0.00	−44.60	0.00	0.00	0.00	Activated
Pathways in cancer	262	107	0.00	−92.70	0.00	0.00	0.00	Activated
Measles	94	54	0.00	−21.80	0.04	0.00	0.00	Activated
Osteoclast differentiation	92	49	0.00	−54.00	0.00	0.00	0.00	Activated
Chemokine signaling pathway	114	50	0.01	−76.50	0.00	0.00	0.00	Activated

Pathways with pGFdr, below 0.0C5 are considered as significant.

Based on the False Discovery Rate (pGFdr) cutoff of 0.05, only three pathways are identified as significant in the NHBE lung cell type (cf., Table 3 and top-left panel in Figure 2), “Cytokine-cytokine receptor interaction” and “Chemokine signaling pathway” are associated with inflammation and immune response (Turner et al., 2014), and are known to be impacted by SARS-CoV-2 infection (Costela-Ruiz et al., 2020). Meanwhile, “Pertussis” is linked to common symptoms of COVID-19 disease, such as paroxysmal coughing with whooping and post-tussive vomiting (De Gouw et al., 2011).

The lung cell type A549 is analyzed to identify the top 10 significant signaling pathways (cf., Table 4 and top-right panel in Figure 2). Among these pathways, “Cytokine-cytokine receptor interaction” is not only the most significant for A549 but also the same as the top one for NHBE. Furthermore, “Rheumatoid arthritis” is a pathway associated with bone destruction (Takayanagi, 2009) and linked to severity and risk of COVID-19 (Dewanjee et al., 2021). Another pathway, “MAPK signaling pathway”, is crucial for various cellular processes such as proliferation, differentiation, development, inflammatory responses, and apoptosis (Zhang and Liu, 2002), and can be provoked by SARS-CoV-2 infection (Grimes and Grimes, 2020).

For the lung cell type A549.ACE2 (cf., Table 5 and bottom-left panel in Figure 2), the top three significant signaling pathways are identified as follows. The “Circadian rhythm—mammal” pathway controls the internal biological clock that enables the sustenance of 24-h physiological and behavioral processes in organisms (Takahashi, 2017), which is shown to be perturbated by SARS-CoV-2 infection (Liu et al., 2021). Landles and Bates (2004) described “Huntington’s disease” as an autosomal-dominant neurodegenerative disorder that primarily affects medium spiny striatal neurons (MSN) and leads to symptoms such as motor dysfunction (e.g., choreiform and involuntary movements), cognitive decline (e.g., dementia), and psychiatric disturbances (e.g., personality changes). “Parkinson’s disease” is a neurodegenerative movement disorder (Abou-Sleiman et al., 2006) that is, related to the third significant signaling pathway. Notably, several recent studies have linked COVID-19 disease to the pathways named after these diseases. (Ellul et al., 2020; Rahman et al., 2021).

Among the top 10 significant signaling pathways identified for the lung cell type Calu3 (cf., Table 6; bottom-right panel in Figure 2), the most significant one is “Influenza A” which is responsible for annual seasonal flu epidemics and periodic pandemics worldwide (Michael et al., 2013) and also related to COVID pandemic (Flerlage et al., 2021). “Cytokine-cytokine receptor interaction” is also identified as a significant signaling pathway for Calu3, similar to NHBE and A549. Additionally, the pathway, “Herpes simplex infection”, is associated with various symptoms, including orofacial lesions, infectious blindness, viral encephalitis, genital lesions, and neonatal encephalitis (Steiner and Benninger, 2013), and can be triggered by COVID-19 infection (Shanshal and Ahmed, 2021).

**FIGURE 3:**
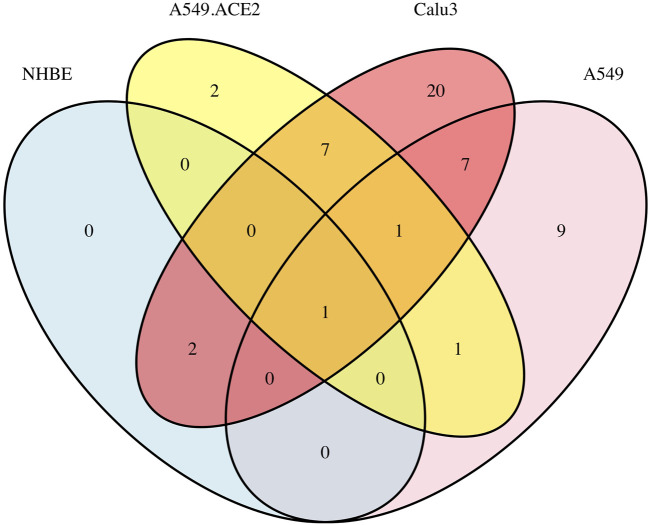
Overlap of significant signaling pathways in four lung cell lines A549, A549.ACE2, Calu3, and NHBE.

Furthermore, Supplementary Figure S5 displays six Venn diagrams that compare the significant signaling pathways shared among the four lung cell lines: A549 vs. NHBE, A549.ACE2 vs. NHBE, Calu3 vs. NHBE, A549 vs. A549.ACE2, A549 vs. Calu3, and A549.ACE2 vs. Calu3. In addition, Figure 3 illustrates the significant signaling pathways that overlap among these four lung cell lines. Only one significant signaling pathway overlaps across these four lung cell lines, which is the “cytokine-cytokine receptor interaction” pathway.

### 3.3 Differential network analysis

DN analysis is conducted on two sets of genes: 1) a total of 13,111 genes and 2) DE genes identified for each lung cell line. The DE genes are obtained using *DESeq2*, and Table 1 shows the number of DE genes for each cell line: 34 for NHBE, 1794 for A549, 7926 for A549.ACE2, and 4266 for Calu3.

To determine the DC pathways that are significantly affected by these genes, we analyze the DN results for both sets of genes separately. For the entire set of 13,111 genes, we identify the top ten significant DC pathways for each lung cell line and these results are presented in Tables 7–10. Meanwhile, for the DE genes, we obtain the top 10 significant DC pathways for each lung cell line and these results are shown in Supplementary Tables S10–S13. The DN analysis results for NHBE and Calu3 show relatively large *p*-values of differential connectivity score for each pathway, which could be attributed to the small sample size of these two cell lines, as each group only has three replicates (mock vs. SARS-CoV-2 infected). By examining the significant DC pathways for both sets of genes, we can gain insight into the specific biological processes that are influenced by these genes in each lung cell line. This information can help us better understand the molecular mechanisms underlying the COVID-19 disease. Based on the outcomes of the DN analysis conducted on 13,111 genes, the subsequent analysis is performed.

**TABLE 7:** Ten most significant DC pathways identified by *dnapath* for the lung cell line NHBE based on a total of 13,111 genes.

Pathway	dc score	*p*-value	n genes	n dc	mean1	mean2
Platelet degranulation	0.02	0.10	129	31	4.25	4.23
Response to elevated platelet cytosolic Ca2+	0.02	0.10	134	30	4.20	4.18
Apoptosis	0.01	0.10	180	31	5.26	5.25
Intrinsic Pathway for Apoptosis	0.04	0.10	53	6	5.38	5.34
Activation of BAD and translocation to mitochondria	0.14	0.10	15	4	6.82	6.81
Activation of BH3-only proteins	0.07	0.10	30	9	5.69	5.63
Signaling by VEGF	0.02	0.10	108	16	5.41	5.40
Negative regulation of the PI3K/AKT network	0.02	0.10	110	21	3.15	3.11
Regulation of gene expression in beta cells	0.18	0.10	21	2	0.589	0.55
VEGFA-VEGFR2 Pathway	0.02	0.10	99	15	5.58	5.57

The columns include the name of the pathway (pathway), differential connectivity score (dc score), the corresponding *p*-value of the dc score (*p*-value), the number of genes on the pathway (n genes), the number of significantly differentially connected genes with *p*-value less than 0.10 on the pathway (n dc), the mean expression of genes in the mock-treated group (mean1), and the mean expression of genes in the SARS-CoV-2-infected group (mean2).

**TABLE 8:** Ten most significant DC pathways identified by *dnapath* for the lung cell line A549 based on a total of 13,111 genes.

Pathway	dc score	*p*-value	n genes	n dc	mean1	mean2
Platelet degranulation	0.03	0.01	129	42	4.18	4.23
Response to elevated platelet cytosolic Ca2+	0.03	0.01	134	45	4.18	4.23
Phase II - Conjugation of compounds	0.05	0.01	107	24	2.67	2.64
Metabolism of nucleotides	0.04	0.01	101	33	4.62	4.59
Intrinsic Pathway for Apoptosis	0.06	0.01	53	17	5.63	5.64
Negative regulation of the PI3K/AKT network	0.04	0.01	110	37	3.30	3.36
PI3K/AKT Signaling in Cancer	0.04	0.01	101	37	3.27	3.29
CD28 dependent PI3K/Akt signaling	0.14	0.01	22	8	4.49	4.44
Constitutive Signaling by AKT1 E17K in Cancer	0.11	0.01	25	11	5.75	5.67
FOXO-mediated transcription	0.06	0.01	65	22	4.52	4.68

The fifth column represents the number of significantly differentially connected genes with *p*-value less than 0.10 on the pathway (n dc).

**TABLE 9:** Ten most significant DC pathways identified by *dnapath* for the lung cell line A549.ACE2 based on a total of 13,111 genes.

Pathway	dc score	*p*-value	n genes	n dc	mean1	mean2
Platelet degranulation	0.0	0.01	129	50	4.30	4.05
Response to elevated platelet cytosolic Ca2+	0.03	0.01	134	47	4.30	4.06
Phase II - Conjugation of compounds	0.06	0.01	107	29	2.52	2.25
Metabolism of nucleotides	0.04	0.01	101	30	4.31	3.97
Cell-Cell communication	0.04	0.01	129	41	3.64	3.49
Regulation of Insulin-like Growth Factor transport and uptake by Insulin-like Growth Factor Binding Proteins	0.04	0.01	125	36	3.67	3.43
Cell-cell junction organization	0.09	0.01	64	19	2.50	2.41
Cell junction organization	0.05	0.01	91	32	3.49	3.30
Myogenesis	0.15	0.01	30	12	3.53	3.54
Post-translational protein phosphorylation	0.04	0.01	108	34	4.02	3.79

The fifth column represents the number of significantly differentially connected genes with *p*-value less than 0.10 on the pathway (n dc).

**TABLE 10:** Ten most significant DC pathways identified by *dnapath* for the lung cell line Calu3 based on a total of 13,111 genes.

Pathway	dc score	*p*-value	n genes	n dc	mean1	mean2
Phase II - Conjugation of compounds	0.03	0.10	107	35	2.58	2.45
Metabolism of nucleotides	0.02	0.10	101	54	4.40	4.19
Purine salvage	0.16	0.10	13	8	4.39	4.26
Nucleotide salvage	0.09	0.10	23	13	4.50	4.50
Cell-Cell communication	0.02	0.10	129	48	3.83	3.95
Regulation of Insulin-like Growth Factor transport and uptake by Insulin-like Growth Factor Binding Proteins	0.02	0.10	125	33	3.32	3.31
Adherens junctions interactions	0.11	0.10	33	8	2.49	2.54
Cell-cell junction organization	0.05	0.10	64	21	2.87	2.89
Cell junction organization	0.03	0.10	91	29	3.81	3.86
Myogenesis	0.09	0.10	30	10	3.08	3.20

The fifth column represents the number of significantly differentially connected genes with *p*-value less than 0.10 on the pathway (n dc).

The lung cell line NHBE infected with SARS-CoV-2 demonstrates three important characteristics (cr., Table 7). First, Coppinger et al. (2004) reported that “platelet degranulation” is associated with vascular injury, and it is known to be dysregulated in response to COVID-19 infection (Zamanian-Azodi et al., 2021). Second, Varga-Szabo et al. (2009) identified that “response to elevated platelet cytosolic Ca2+” serves as an event of platelet degranulation, which is essential for platelet activation in hemostasis and thrombosis. Third, MacFarlane and Williams (2004) revealed that “apoptosis” is related to cell death and manipulated by SARS-CoV-2 virus to evade its own elimination (Li et al., 2022).

For the lung cell line A549 (cr., Table 8), the two most significant DC pathways identified from DN analysis are similar to NHBE, which are “Platelets degranulation” and “Response to elevated platelet cytosolic Ca2+”. Additionally, the pathway “Phase II - Conjugation of compounds” is found to play a crucial role in the detoxification of various xenobiotics, drug metabolism, and endogenous substrates (Jančová and Šiller, 2012) and be suppressed by the COVID-19 disease (Hammoudeh et al., 2021).

For the lung cell line A549.ACE2 (cr., Table 9), similar to A549, the top three significant DC pathways identified from DN analysis are “Platelets degranulation”, “Response to elevated platelet cytosolic Ca2+”, and “Phase II—Conjugation of compounds”.

For the lung cell line Calu3 (cr., Table 10), the top significant DC pathway identified from DN analysis is “Phase II—Conjugation of compounds”, which is also significant for A549 and A549.ACE2. In addition, two other DC pathways are also identified as significant. “Metabolism of nucleotides” is essential to cellular signaling and energy transduction events (Welin and Nordlund, 2010) and affected by SARS-CoV-2 virus (Qin et al., 2022). “Purine salvage” plays a crucial role in energy conservation (Cappiello et al., 1992), and it is closely related to SARS-CoV-2 replication (Zhang et al., 2021b).

**FIGURE 4:**
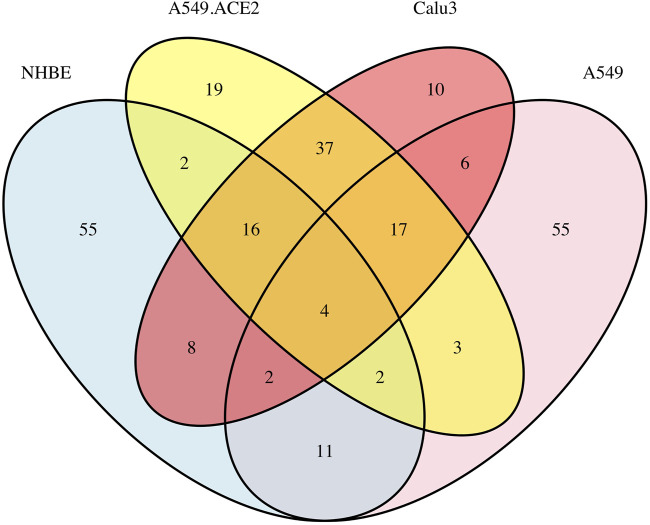
Overlap of the top 100 significant pathways based on the DN analysis of the four lung cell lines: A549, A549.ACE2, Calu3, and NHBE, using a total of 13,111 genes.

Figure 4 displays a Venn diagram of the top 100 significant DC pathways resulting from the DN analysis for the four different lung cell lines NHBE, A549, A549.ACE2, and Calu3. The overlapping significant DC pathways identified among these cell lines are “Intrinsic Pathway for Apoptosis”, “Negative regulation of the PI3K/AKT network”, “Fatty acid metabolism”, and “DDX58/IFIH1-mediated induction of interferon-alpha/beta”. “Intrinsic Pathway for Apoptosis” is mainly linked to immune cell depletion during COVID-19 disease (Bader et al., 2022) and triggered by cellular damage or stress, leading to the activation of “apoptosis” pathway (Krüger and Richter, 2022). “Negative regulation of the PI3K/AKT network” is associated with growth factors and hormones (Shorning et al., 2020), and it is known to be activated by SARS-CoV-2 infections (Farahani et al., 2022). “Fatty acid metabolism” is a crucial component of human energy metabolism (Vance and Vance, 1996)and is suggested as a potential target for inhibiting SARS-CoV-2 virus replication (Tanner and Alfieri, 2021). Finally, “DDX58/IFIH1-mediated induction of interferon-alpha/beta”, whose production is influenced by SARS-CoV-2 virus (Feizollahi et al., 2023), is also linked to Influenza A viruses and Hepatitis C Viruses (Yoneyama and Fujita, 2007).

**FIGURE 5:**
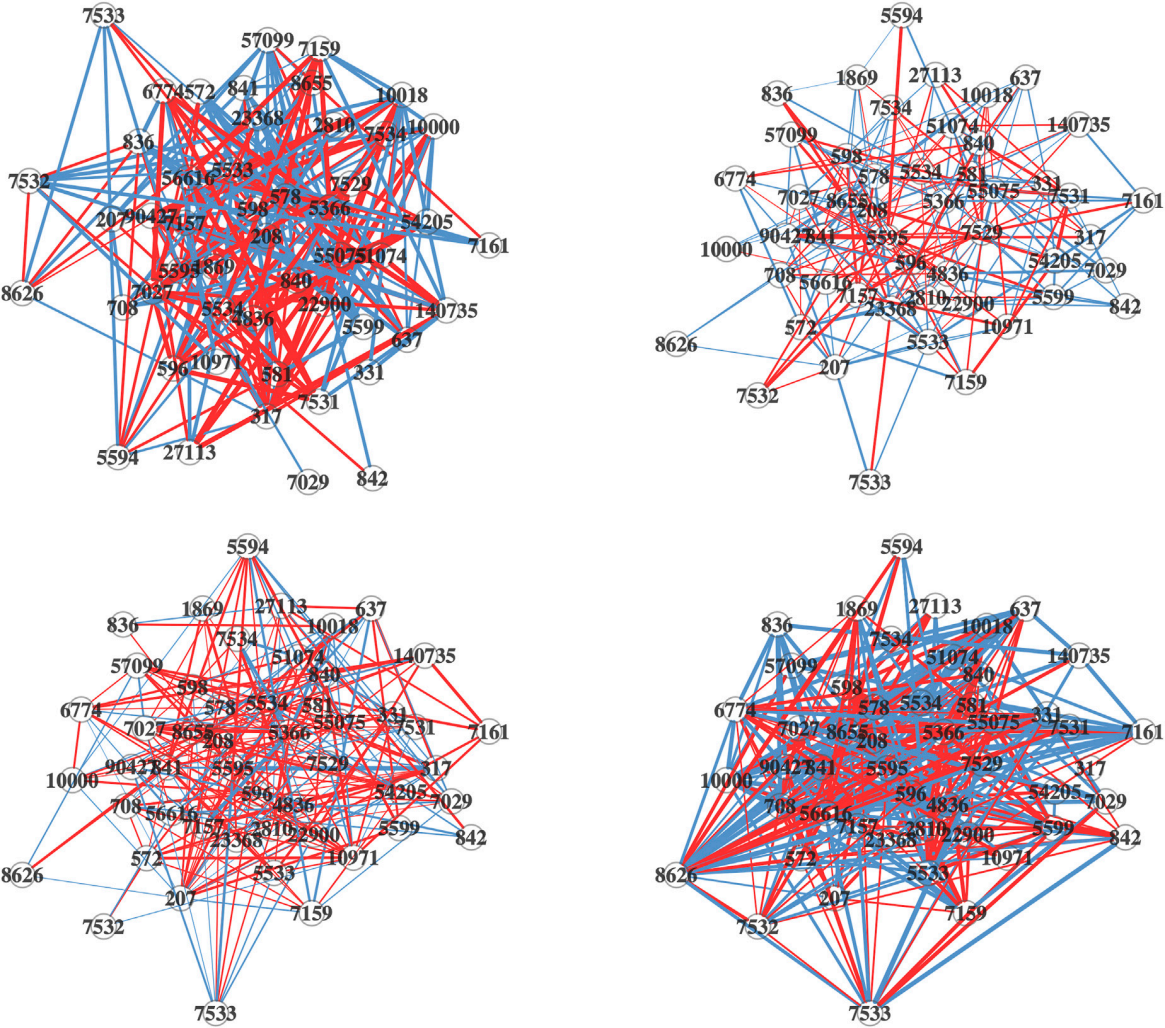
Differential network plots of “Intrinsic Pathway for Apoptosis” for the four lung cell Lines, NHBE (top-left), A549 (top-right), A549.ACE (bottom-left), and Calu3 (bottom-right). In the network plots, nodes represent genes. The edges in the network are color-coded: a blue edge denotes a stronger gene-gene association in the mock-treated group, while a red edge denotes a stronger association in the SARS-CoV-2 infected group. The width of the edges corresponds to the magnitude of the association, and the transparency is determined by the *p*-value of the edge’s differential connectivity score.

The network plots (cr. Figure 5) are generated for each of the four lung cell lines, depicting the differential connectivity of the most significant DC pathway that they share, “Intrinsic Pathway for Apoptosis”. This allows us to visually compare the differential connectivity of this pathway between the different cell lines. As depicted in Figure 5, there is a considerable variation in the differential connectivity of the “Intrinsic Pathway for Apoptosis” across the four lung cell lines. The detailed information about the nodes and edges of this pathway is available for reference in Supplementary Tables S14–S21.

Unfortunately, Reactome does not currently have an exact mapping file to KEGG. Domingo-Fernández et al. (2018) provided insights on the mapping across different pathway databases, introducing a method called ComPath. For the KEGG pathway “cytokine-cytokine receptor interaction”, which is the only pathway found to be significant among all four cell lines, ComPath was used to obtain three sub-pathways from Reactome: “IL-6-type cytokine receptor ligand interactions”, “TNFs bind their physiological receptors”, and “Chemokine receptors bind chemokines”. Among these, “IL-6-type cytokine receptor ligand interactions” was identified as significantly differentially connected for Calu3, “TNFs bind their physiological receptors” for A549, A549.ACE2, and Calu3, and “Chemokine receptors bind chemokines” for NHBE and A549.

## 4 Discussion

The purpose of this study is to analyze the RNA-seq data of the four lung cell lines NHBE, A549, A549.ACE2, and Calu3, and identify their common and unique biological features by conducting the DE analysis, pathway analysis, and DN analysis on the data from each cell line.

This study reveals the shared and unique features of DE genes in the four lung cell lines NHBE, A549, A549.ACE2, and Calu3, using the R packages *DESeq2*, *edgeR*, and *limma*. The cell line A549.ACE2 exhibits the highest number of DE genes, while the cell line NHBE has the lowest, which is consistent with the finding in the literature (Alipoor et al., 2021). Although there exists some variation in the results of DE analysis between our study and other studies, this might be due to differences in data pre-processing and DE analysis methods. The ordered list of the top 10 DE genes for each lung cell line is determined by *RankAggreg*, which combines the results of the three DE analysis methods. Among the DE genes identified for the four lung cell lines, 12 genes are overlapped, which are SAA2, BIRC3, TNFAIP2, IL32, CSF3, C1QTNF1, C3, KYNU, CXCL5, CXCL3, VNN1, and SOD2. These genes are known to be associated with COVID-19 disease, which are elucidated in the recent literature (Malle et al., 2009; Chua et al., 2020; Liang et al., 2020; Menon et al., 2020; Nunnari et al., 2020; Yang et al., 2020; Fang et al., 2021; Zinellu and Mangoni, 2021; Bizjak et al., 2022; Jerotic et al., 2022; Luo et al., 2022; Zamani et al., 2022; Zoodsma et al., 2022). Out of these twelve genes, SAA2, BIRC3, TNFAIP2, IL32, CSF3, C3, KYNU, CXCL5, CXCL3, and SOD2 are linked to inflammation. CSF3, KYNU, and VNN1 are related to the immune system, while C1QTNF1, C3, and SOD2 are associated with blood coagulation. Identifying these specific genes associated with COVID-19 is essential to understand the molecular mechanisms underlying the disease, which can help researchers develop potential drug targets for therapeutic interventions to reduce the severity of COVID-19 and improve patient outcomes.

The study employs *SPIA* to investigate the significant signaling pathways in each of the four lung cell lines—NHBE, A549, A549.ACE2, and Calu3. The number of identified significant pathways by *SPIA* for the four cell lines are 3, 19, 12, and 38, respectively. The results show that the most significant signaling pathways vary among the four cell lines. For NHBE, the most significant signaling pathways are related to inflammation, immune response, and COVID-19 symptoms, such as coughing and vomiting. For A549, the most significant pathways are not only related to inflammation but also to bone destruction. The most significant pathways for A549.ACE2 are associated with internal biological clock, motor dysfunction, cognitive decline, psychiatric disturbances, and neurodegenerative movement disorder. In contrast, the most significant pathways for Calu3 are related to flu epidemics, inflammation, immune system, and orofacial lesions. Interestingly, only one pathway, “cytokine-cytokine receptor interaction” is found to be significant among all four cell lines and is closely related to inflammation and immune response. This finding is consistent with some recent research (Chen et al., 2020; Mehta et al., 2020), and there is a study even summarizes the crucial role that the “cytokine-cytokine receptor interaction” plays in COVID-19 disease (Costela-Ruiz et al., 2020). This information can help researchers better understand the underlying mechanisms of COVID-19 and develop more effective treatment plans that target the specific pathways involved in the disease.

Based on the results of DN analysis by using *dnapath*, it is observed that there are certain biological traits shared and distinct among the four distinct lung cell lines. The DN analysis indicates that the three most significant DC pathways for NHBE are linked to vascular injury and cell death. In contrast, the top three significant DC pathways for A549 are not only associated with vascular injury but also with various detoxification processes. Interestingly, the top three significant DC pathways for A549.ACE2 exhibit functions similar to those of A549. Finally, for Calu3, the three most significant DC pathways are involved in detoxification processes, energy transduction, and energy conservation. The DN analysis reveals that among the 100 most significant DC pathways for each of the four lung cell lines, there are some shared DC pathways, including “Intrinsic Pathway for Apoptosis”, “Negative regulation of the PI3K/AKT network”, “Fatty acid metabolism”, and “DDX58/IFIH1-mediated induction of interferon-alpha/beta”. These pathways are associated with various cellular processes such as cellular damage, growth factors, hormones, energy metabolism, and the periodic occurrence of flu epidemics. Despite the identification of many common significant pathways among the four lung cell lines from the DN analysis, the differential connectivity of these pathways is significantly different among the four cell lines. For instance, the “Intrinsic Pathway for Apoptosis” exhibits distinct differential connectivity patterns among these cell lines. Our results of the DN analysis can provide a more comprehensive understanding of molecular pathways and differential networks that are disrupted in COVID-19 and give potential therapeutic recommendations.

Our study aims to investigate the unique and shared mechanisms underlying COVID-19 disease in the four distinct lung cell lines NHBE, A549, A549.ACE2, and Calu3 using DE analysis, pathway analysis, and DN analysis. Following the application of the various statistical techniques to the RNA-Seq data, we successfully 1) generate the ordered lists of DE genes for individual cell lines through a rank aggregation method, identify overlapping DE genes across four distinct cell lines, 2) discover significant signaling pathways unique to each cell line, recognized shared significant signaling pathways among all cell lines, 3) unveil significant DN pathways for each cell line and identify common significant DC pathways among them. The outcomes of these analyses, which are previously demonstrated, reveal promising results. We eagerly anticipate the validation of these findings by experts in the fields of biology, medicine, and pharmacology. However, our study has limitations, including a small sample size and the absence of covariates in the analysis. Additionally, the current versions of the R packages *SPIA* and *dnapath* utilize different databases for pathway analysis and DN analysis. The former only includes KEGG pathways data, while the latter only includes Reactome pathways data. To address these limitations, we suggest searching more related RNA-seq data, incorporating covariates if clinical data are also available, and trying more approaches of pathway analysis and DN analysis to validate the results and avoid bias caused by relying on only one approach. Integrating both KEGG pathways and Reactome pathways data into these R packages (e.g., *SPIA* and *dnapath*) in the future would enable us to establish connections between our results of pathway analysis and DN analysis. Furthermore, we can also apply similar DE analysis, pathway analysis, and DN analysis to investigate other lung-related diseases using these lung cell lines. Furthermore, we can investigate the distinct and shared mechanisms of the SARS-CoV-2 virus and other comparable viruses, which can provide more insights into the pathogenesis of COVID-19.

## Data Availability

The RNA-Seq data was obtained from the Gene Expression Omnibus (GEO) under the accession number GSE147507 (Blanco-Melo et al., 2020).
