# Supplementary material for: Identification of shared biological features in four different lung cell lines infected with SARS-CoV-2 virus through RNA-seq analysis
Source: Front Genet. 2023 Aug 16;14:1235927. doi: 10.3389/fgene.2023.1235927 (PMC10468990; doi:10.3389/fgene.2023.1235927)
Supplement: Supplementary file 1 [file Table1.pdf]

Supplementary Material

1 DIFFERENTIAL EXPRESSION (DE) ANALYSIS

1.1 DE Genes Comparision between SARS-CoV-2 with and without Rux(Ruxolitinib) Pre-treatment

Supplementary Table 1. Ten most DE genes from the comparison between the mock-treated group and the SARS-CoV-2-infected group with treatment Rux for A549.ACE2 cell line

	basemean	log2FoldChange	lfcSE	stat	pvalue	padj
HIST2H2BE	686.300	4.09665	0.192133	21.3220	7.09946e-101	9.30810e-97
DUSP8	557.724	4.31084	0.206853	20.8401	1.87493e-96	1.22911e-92
EGR1	888.602	7.49075	0.362883	20.6423	1.14420e-94	5.00054e-91
IER5	1694.540	2.67076	0.131792	20.2650	2.61995e-91	8.58753e-88
PCF11	1269.831	2.77730	0.139057	19.9724	9.57972e-89	2.51199e-85
BCL3	796.448	2.95421	0.155316	19.0207	1.14990e-80	2.51271e-77
NR1D1	955.825	5.69377	0.299543	19.0082	1.45936e-80	2.73338e-77
NFKBIA	6498.537	4.96419	0.264306	18.7819	1.06126e-78	1.73927e-75
NFKBIE	355.995	3.60530	0.194950	18.4935	2.33018e-76	3.39456e-73
CCNL1	2604.374	3.86340	0.217127	17.7933	7.97136e-71	1.04513e-67

The columns consist of several variables for each gene, including the base means across samples (basemean), log2 fold changes (log2FoldChange), standard errors of log2 fold changes (lfcSE), Wald test statistics (stat), p-values corresponding to the Wald test statistics (pvalue), and FDR-adjusted p-values (padj).

Supplementary Table 2. Ten most DE genes from the comparison between the mock-treated group and the SARS-CoV-2-infected group without treatment Rux for A549.ACE2 cell line

	basemean	log2FoldChange	lfcSE	stat	pvalue	padj
EGR1	888.602	7.93639	0.321907	24.6543	3.31073e-134	4.34069e-130
IER5	1694.540	2.57180	0.109305	23.5285	2.08188e-122	1.36477e-118
HIST2H2BE	686.300	3.78138	0.161402	23.4284	2.19670e-121	9.60032e-118
DUSP8	557.724	3.97228	0.174837	22.7199	2.84642e-114	9.32984e-111
PCF11	1269.831	2.50539	0.115988	21.6004	1.78100e-103	4.67013e-100
NFKBIE	355.995	3.58977	0.169966	21.1205	5.15073e-99	1.12552e-95
BCL3	796.448	2.72412	0.130857	20.8176	2.99736e-96	5.61405e-93
NFKBIA	6498.537	4.43889	0.216523	20.5008	2.11821e-93	3.47148e-90
NR1D1	955.825	4.95828	0.250237	19.8144	2.23830e-87	3.26070e-84
JUNB	1978.670	3.51680	0.180966	19.4335	4.01657e-84	5.26613e-81

1.2 DE Genes Comparision between A549 Cell Line and A549.ACE2 Cell Line

Supplementary Table 3. Ten most DE genes from the comparison between the mock-treated group and the SARS-CoV-2-infected group for A549 cell line

	basemean	log2FoldChange	lfcSE	stat	pvalue	padj
BTG3	440.742	0.603093	0.0901728	6.68819	2.25941e-11	2.84730e-07
TXNIP	2086.842	5.268171	0.8443706	6.23917	4.39903e-10	1.38591e-06
SERPINB7	176.620	2.335833	0.3710269	6.29559	3.06235e-10	1.38591e-06
CLDN1	2093.871	1.155840	0.1851109	6.24404	4.26404e-10	1.38591e-06
LAMC2	19528.233	2.286555	0.3695141	6.18801	6.09301e-10	1.53568e-06
GAS5	1481.716	1.990948	0.3276130	6.07713	1.22351e-09	2.20266e-06
ANXA3	1860.661	0.730404	0.1199372	6.08989	1.12991e-09	2.20266e-06
EREG	1864.821	1.969075	0.3426594	5.74645	9.11372e-09	1.43564e-05
ASNS	739.601	2.223928	0.3894386	5.71060	1.12579e-08	1.57636e-05
DNAJC21	439.534	0.472492	0.0840994	5.61826	1.92890e-08	2.43080e-05

Supplementary Table 4. Ten most DE genes from the comparison between the mock-treated group and the SARS-CoV-2-infected group for A549.ACE2 cell line

	basemean	log2FoldChange	lfcSE	stat	pvalue	padj
EGR1	888.602	7.93639	0.321907	24.6543	3.31073e-134	4.34069e-130
IER5	1694.540	2.57180	0.109305	23.5285	2.08188e-122	1.36477e-118
HIST2H2BE	686.300	3.78138	0.161402	23.4284	2.19670e-121	9.60032e-118
DUSP8	557.724	3.97228	0.174837	22.7199	2.84642e-114	9.32984e-111
PCF11	1269.831	2.50539	0.115988	21.6004	1.78100e-103	4.67013e-100
NFKBIE	355.995	3.58977	0.169966	21.1205	5.15073e-99	1.12552e-95
BCL3	796.448	2.72412	0.130857	20.8176	2.99736e-96	5.61405e-93
NFKBIA	6498.537	4.43889	0.216523	20.5008	2.11821e-93	3.47148e-90
NR1D1	955.825	4.95828	0.250237	19.8144	2.23830e-87	3.26070e-84
JUNB	1978.670	3.51680	0.180966	19.4335	4.01657e-84	5.26613e-81

1.3 DE Genes Comparision the Mock-treated Group and the SARS-CoV-2-infected Group for the four cell lines

Supplementary Table 5. Ten most DE genes from the comparison between the mock-treated group and the SARS-CoV-2-infected group for NHBE cell line

	basemean	log2FoldChange	lfcSE	stat	pvalue	padj
CXCL5	11013.0246	3.498609	0.362155	9.66052	4.43595e-22	5.81598e-18
PLAT	829.4167	1.394660	0.159280	8.75603	2.02255e-18	1.32588e-14
CSF3	21.4742	4.849140	0.564818	8.58532	9.05858e-18	3.95890e-14
TNFAIP2	6165.2873	1.550246	0.182072	8.51445	1.67377e-17	5.48621e-14
ZC3H12A	1086.0169	1.672972	0.230558	7.25620	3.98115e-13	1.04394e-09
IFITM10	84.3061	-1.571569	0.217711	-7.21861	5.25217e-13	1.14769e-09
BPGM	355.4230	0.961909	0.155670	6.17916	6.44442e-10	1.20704e-06
OLFML2A	191.9710	-0.952542	0.154978	-6.14629	7.93138e-10	1.29985e-06
TNIP1	2796.0332	1.264196	0.244674	5.16686	2.38064e-07	3.46806e-04
KYNU	2282.5747	0.847817	0.164960	5.13954	2.75411e-07	3.61092e-04

Supplementary Table 6. Ten most DE genes from the comparison between the mock-treated group and the SARS-CoV-2-infected group for A549 cell line

	basemean	log2FoldChange	lfcSE	stat	pvalue	padj
BTG3	440.7424	0.602887	0.0913233	6.60168	4.06517e-11	5.22618e-07
CLDN1	2093.8708	1.155846	0.1845058	6.26455	3.73905e-10	1.60231e-06
TXNIP	2086.8423	5.267527	0.8356404	6.30358	2.90845e-10	1.60231e-06
ANXA3	1860.6612	0.730448	0.1185866	6.15962	7.29202e-10	2.07314e-06
GAS5	1481.7163	1.990945	0.3256013	6.11467	9.67553e-10	2.07314e-06
SERPINB7	176.6200	2.335608	0.3805891	6.13682	8.41882e-10	2.07314e-06
LAMC2	19528.2331	2.286529	0.3757550	6.08516	1.16375e-09	2.13731e-06
ADM2	68.4037	3.449348	0.5799091	5.94808	2.71300e-09	4.35979e-06
NEB	114.6702	-1.831791	0.3272533	-5.59747	2.17502e-08	2.79620e-05
EREG	1864.8212	1.969013	0.3509546	5.61045	2.01803e-08	2.79620e-05

Supplementary Table 7. Ten most DE genes from the comparison between the mock-treated group and the SARS-CoV-2-infected group for A549.ACE2 cell line

	basemean	log2FoldChange	lfcSE	stat	pvalue	padj
IER5	1694.540	2.60609	0.0994467	26.2059	2.27509e-151	2.98286e-147
HIST2H2BE	686.300	3.89607	0.1538334	25.3265	1.63062e-141	1.06895e-137
EGR1	888.602	7.79389	0.3160870	24.6574	3.06380e-134	1.33898e-130
DUSP8	557.724	4.09383	0.1664664	24.5925	1.51847e-133	4.97715e-130
PCF11	1269.831	2.60335	0.1110021	23.4531	1.22776e-121	3.21944e-118
NFKBIE	355.995	3.59513	0.1561140	23.0289	2.39657e-117	5.23690e-114
BCL3	796.448	2.80642	0.1225782	22.8950	5.21548e-116	9.76859e-113
NFKBIA	6498.537	4.63619	0.2073720	22.3569	1.03513e-110	1.69644e-107
ZC3H4	660.895	2.01724	0.0940318	21.4528	4.30105e-102	6.26567e-99
CCNL1	2604.374	3.60470	0.1701315	21.1877	1.23897e-99	1.62442e-96

Supplementary Table 8. Ten most DE genes from the comparison between the mock-treated group and the SARS-CoV-2-infected group for Calu3 cell line

	basemean	log2FoldChange	lfcSE	stat	pvalue	padj
THBS1	4180.4978	2.65034	0.1257974	21.0683	1.55363e-98	2.03697e-94
DAPP1	223.8687	2.44959	0.1241658	19.7283	1.23124e-86	8.07142e-83
TNFAIP2	6165.2873	3.21070	0.1778271	18.0552	7.18633e-73	3.14067e-69
ADRB2	182.8626	2.46296	0.1463614	16.8279	1.52289e-63	4.99166e-60
PLAT	829.4167	2.44228	0.1471356	16.5988	7.10762e-62	1.86376e-58
MAP3K8	572.6413	2.82592	0.1758265	16.0722	3.99734e-58	7.48702e-55
NUAK2	990.2233	4.37313	0.2720706	16.0735	3.91299e-58	7.48702e-55
B4GALT5	1915.9902	1.42747	0.0945716	15.0940	1.77310e-51	2.90588e-48
TNF	95.9042	7.54483	0.5418931	13.9231	4.58581e-44	6.68051e-41
IRS2	360.4361	2.24617	0.1746950	12.8576	7.79106e-38	1.02149e-34

1.4 Numbers of up-regulated and down-regulated DE genes in the four lung cell lines (NHBE, A549, A549.ACE2, and Calu3) using *DESeq2*.

Supplementary Table 9. Numbers of up-regulated and down-regulated DE genes in the four lung cell lines (NHBE, A549, A549.ACE2, and Calu3) using *DESeq2*

	NHBE	A549	A549.ACE2	Calu3
up-regulated	29	1139	4210	2207
down-regulated	5	655	3716	2059

2 DIFFERENTIAL NETWORK (DN) ANALYSIS

2.1 Significant Differentially Connected (DC) Pathways from DN analysis Based on DE Genes

Supplementary Table 10. Ten most significant DC pathways identified by *dnapath* for the lung cell line NHBE based on DE genes.

pathway	dc score	p-value	n genes	n dc	mean1	mean2
Chemokine receptors bind chemokines	3.54e- 1	0.1	59	4	0.146	0.275
Interleukin-4 and Interleukin-13 signaling	5.55e-16	0.4	108	0	0.0785	0.110
Toll Like Receptor 4 (TLR4) Cascade	5.55e-16	0.6	131	0	0.0393	0.0593
MyD88-independent TLR4 cascade	5.55e-16	0.6	99	0	0.0520	0.0784
Toll Like Receptor 3 (TLR3) Cascade	5.55e-16	0.6	94	0	0.0548	0.0826
Toll-like Receptor Cascades	5.55e-16	0.6	158	0	0.0326	0.0491
TRIF(TICAM1)-mediated TLR4 signaling	5.55e-16	0.6	99	0	0.0520	0.0784
Interleukin-10 signaling	7.5 e- 1	0.6	47	0	0.0911	0.211
Programmed Cell Death	0	NA	196	0	0.0395	0.0503
Other interleukin signaling	0	NA	24	0	0.173	0.405

The columns include the name of the pathway (pathway), differential connectivity score (dc score), the corresponding p-value of the dc score (p-value), the number of genes on the pathway (n genes), the number of significantly differentially connected genes with p-value less than 0.10 on the pathway (n dc), the mean expression of genes in the mock-treated group (mean1), and the mean expression of genes in the SARS-CoV-2-infected group (mean2).

Supplementary Table 11. Ten most significant DC pathways identified by *dnapath* for the lung cell line A549 based on DE genes.

pathway	dc score	p-value	n genes	n dc	mean1	mean2
Platelet degranulation	0.161	0.00990	129	14	0.945	0.980
Response to elevated platelet cytosolic Ca2+	0.154	0.00990	134	16	0.922	0.959
Phase II - Conjugation of compounds	0.423	0.00990	107	4	0.193	0.213
Metabolism of nucleotides	0.196	0.00990	101	8	0.737	0.793
Regulation of Insulin-like Growth Factor (IGF) transport and uptake by Insulin-li…	0.212	0.00990	125	10	0.578	0.661
Post-translational protein phosphorylation	0.223	0.00990	108	6	0.609	0.684
Apoptosis	0.106	0.00990	180	7	0.934	0.939
PI3K/AKT Signaling in Cancer	0.131	0.00990	101	12	1.07	1.11
Costimulation by the CD28 family	0.284	0.00990	69	6	0.721	0.739
CD28 co-stimulation	0.284	0.00990	33	6	1.51	1.54

The fifth column represents the number of significantly differentially connected genes with p-value less than 0.10 on the pathway (n dc).

Supplementary Table 12. Ten most significant DC pathways identified by *dnapath* for the lung cell line A549.ACE2 based on DE genes.

pathway	dc score	p-value	n genes	n dc	mean1	mean2
Platelet degranulation	0.0554	0.00990	129	30	3.22	2.98
Response to elevated platelet cytosolic Ca2+	0.0524	0.00990	134	32	3.19	2.97
Phase II - Conjugation of compounds	0.0880	0.00990	107	11	1.85	1.61
Metabolism of nucleotides	0.0567	0.00990	101	19	3.23	2.92
Cell-Cell communication	0.0591	0.00990	129	16	2.52	2.43
Regulation of Insulin-like Growth Factor (IGF) transport and uptake by Insulin-li…	0.0592	0.00990	125	26	2.66	2.43
Cell junction organization	0.0843	0.00990	91	15	2.52	2.37
Post-translational protein phosphorylation	0.0622	0.00990	108	24	2.95	2.71
Apoptosis	0.0336	0.00990	180	47	3.69	3.62
Intrinsic Pathway for Apoptosis	0.0952	0.00990	53	12	3.86	3.95

The fifth column represents the number of significantly differentially connected genes with p-value less than 0.10 on the pathway (n dc).

Supplementary Table 13. Ten most significant DC pathways identified by *dnapath* for the lung cell line Calu3 based on DE genes.

pathway	dc score	p-value	n genes	n dc	mean1	mean2
Platelet degranulation	0.0758	0.1	129	23	1.27	1.34
Response to elevated platelet cytosolic Ca2+	0.0724	0.1	134	24	1.26	1.33
Phase II - Conjugation of compounds	0.110	0.1	107	10	0.835	0.779
Metabolism of nucleotides	0.0602	0.1	101	24	1.66	1.56
Purine salvage	0.435	0.1	13	3	1.53	1.36
Nucleotide salvage	0.259	0.1	23	3	1.54	1.53
Cell-Cell communication	0.0709	0.1	129	22	1.10	1.17
Regulation of Insulin-like Growth Factor (IGF) transport and uptake by Insulin-li...	0.0812	0.1	125	13	0.919	0.949
Adherens junctions interactions	0.355	0.1	33	4	0.815	0.829
Cell-cell junction organization	0.179	0.1	64	8	0.764	0.745

The fifth column represents the number of significantly differentially connected genes with p-value less than 0.10 on the pathway (n dc).

2.2 Node Information of “Intrinsic Pathway for Apoptosis” for each Cell Line

Supplementary Table 14. Ten most significant DC nodes identified by *dnapath* for the lung cell line NHBE.

pathway	genes	dc score	p-value	mean1	mean2
Intrinsic Pathway for Apoptosis	6774	0.00210	0.1	7.35	7.47
Intrinsic Pathway for Apoptosis	5533	0.00209	0.1	4.95	4.81
Intrinsic Pathway for Apoptosis	7159	0.00192	0.1	5.92	6.00
Intrinsic Pathway for Apoptosis	140735	0.00180	0.1	5.62	5.49
Intrinsic Pathway for Apoptosis	10018	0.00180	0.1	3.57	3.01
Intrinsic Pathway for Apoptosis	317	0.00154	0.1	5.02	4.82
Intrinsic Pathway for Apoptosis	56616	0.00186	0.2	5.53	5.50
Intrinsic Pathway for Apoptosis	10000	0.00180	0.2	4.87	4.65
Intrinsic Pathway for Apoptosis	836	0.00163	0.2	5.41	5.31
Intrinsic Pathway for Apoptosis	90427	0.00214	0.3	1.55	1.44

Supplementary Table 15. Ten most significant DC nodes identified by *dnapath* for the lung cell line A549.

pathway	genes	dc score	p-value	mean1	mean2
Intrinsic Pathway for Apoptosis	840	0.00603	0.00990	5.40	5.65
Intrinsic Pathway for Apoptosis	7529	0.00546	0.00990	8.27	8.40
Intrinsic Pathway for Apoptosis	90427	0.00531	0.00990	3.31	3.61
Intrinsic Pathway for Apoptosis	5533	0.00426	0.00990	3.64	4.10
Intrinsic Pathway for Apoptosis	207	0.00387	0.00990	7.65	7.22
Intrinsic Pathway for Apoptosis	55075	0.00332	0.00990	6.83	7.51
Intrinsic Pathway for Apoptosis	5366	0.00303	0.00990	3.52	4.30
Intrinsic Pathway for Apoptosis	4836	0.00281	0.00990	7.76	7.55
Intrinsic Pathway for Apoptosis	5595	0.00648	0.0198	5.68	5.67
Intrinsic Pathway for Apoptosis	581	0.00324	0.0198	7.32	6.83

Supplementary Table 16. Ten most significant DC nodes identified by *dnapath* for the lung cell line A549.ACE2.

pathway	genes	dc score	p-value	mean1	mean2
Intrinsic Pathway for Apoptosis	5366	0.00839	0.00990	5.41	7.07
Intrinsic Pathway for Apoptosis	7157	0.00829	0.00990	6.62	6.18
Intrinsic Pathway for Apoptosis	10018	0.00813	0.00990	4.07	4.97
Intrinsic Pathway for Apoptosis	317	0.00727	0.00990	5.42	6.56
Intrinsic Pathway for Apoptosis	637	0.00662	0.00990	5.74	5.28
Intrinsic Pathway for Apoptosis	10971	0.00592	0.00990	9.43	8.49
Intrinsic Pathway for Apoptosis	5595	0.00573	0.00990	4.57	3.76
Intrinsic Pathway for Apoptosis	7027	0.00572	0.00990	6.37	5.58
Intrinsic Pathway for Apoptosis	207	0.00548	0.00990	7.02	6.53
Intrinsic Pathway for Apoptosis	57099	0.00528	0.00990	4.84	4.29

Supplementary Table 17. Ten most significant DC nodes identified by *dnapath* for the lung cell line Calu3.

pathway	genes	dc score	p-value	mean1	mean2
Intrinsic Pathway for Apoptosis	90427	0.00226	0.1	2.57	3.65
Intrinsic Pathway for Apoptosis	8626	0.00223	0.1	-2.49	-1.06
Intrinsic Pathway for Apoptosis	8655	0.00195	0.1	8.57	7.91
Intrinsic Pathway for Apoptosis	10000	0.00195	0.1	6.23	7.08
Intrinsic Pathway for Apoptosis	56616	0.00193	0.1	6.24	5.99
Intrinsic Pathway for Apoptosis	5599	0.00185	0.1	4.14	4.80
Intrinsic Pathway for Apoptosis	637	0.00150	0.1	7.23	6.65
Intrinsic Pathway for Apoptosis	6774	0.00148	0.1	7.50	7.93
Intrinsic Pathway for Apoptosis	208	0.00148	0.1	7.49	7.00
Intrinsic Pathway for Apoptosis	708	0.00148	0.1	6.77	6.28

2.3 Edge Information of “Intrinsic Pathway for Apoptosis” for each Cell Line

Supplementary Table 18. Ten most significant DC edges identified by *dnapath* for the lung cell line NHBE.

pathway	edges	dc score	p-value	nw1	nw2
Intrinsic Pathway for Apoptosis	207 - 57099	0.00432	0.1	0.0357	-0.0300
Intrinsic Pathway for Apoptosis	207 - 581	0.00259	0.1	-0.0209	0.0300
Intrinsic Pathway for Apoptosis	207 - 708	0.00432	0.1	0.0357	-0.0300
Intrinsic Pathway for Apoptosis	207 - 841	0.00432	0.1	0.0357	-0.0300
Intrinsic Pathway for Apoptosis	207 - 5366	0.00245	0.1	-0.0357	0.0139
Intrinsic Pathway for Apoptosis	208 - 317	0.00140	0.1	0.0209	-0.0165
Intrinsic Pathway for Apoptosis	208 - 51074	0.00400	0.1	-0.0468	0.0165
Intrinsic Pathway for Apoptosis	208 - 572	0.000279	0.1	0.0468	0.0300
Intrinsic Pathway for Apoptosis	208 - 598	0.00590	0.1	-0.0468	0.0300
Intrinsic Pathway for Apoptosis	208 - 637	0.00590	0.1	-0.0468	0.0300

nw1 and nw2 represent the estimated association in the mock-treated and SARS-CoV-2 infected groups.

Supplementary Table 19. Ten most significant DC edges identified by *dnapath* for the lung cell line A549.

pathway	genes	dc score	p-value	nw1	nw2
Intrinsic Pathway for Apoptosis	207 - 90427	0.0223	0.00990	0.0843	-0.0652
Intrinsic Pathway for Apoptosis	207 - 5533	0.0289	0.00990	-0.183	-0.0130
Intrinsic Pathway for Apoptosis	208 - 8626	0.0123	0.00990	0.0584	-0.0527
Intrinsic Pathway for Apoptosis	51074 - 56616	0.00449	0.00990	0.0219	0.0890
Intrinsic Pathway for Apoptosis	51074 - 5366	0.0155	0.00990	-0.0638	0.0605
Intrinsic Pathway for Apoptosis	51074 - 55075	0.00647	0.00990	-0.0583	0.0222
Intrinsic Pathway for Apoptosis	51074 - 7529	0.0180	0.00990	-0.0407	0.0935
Intrinsic Pathway for Apoptosis	57099 - 55075	0.0175	0.00990	0.0416	-0.0906
Intrinsic Pathway for Apoptosis	572 - 6774	0.0226	0.00990	-0.0861	0.0642
Intrinsic Pathway for Apoptosis	578 - 10018	0.0166	0.00990	0.0972	-0.0316

Supplementary Table 20. Ten most significant DC edges identified by *dnapath* for the lung cell line A549.ACE2.

pathway	genes	dc score	p-value	nw1	nw2
Intrinsic Pathway for Apoptosis	207 - 208	0.00838	0.00990	-0.0425	0.0490
Intrinsic Pathway for Apoptosis	207 - 51074	0.0266	0.00990	0.0442	-0.119
Intrinsic Pathway for Apoptosis	207 - 8655	0.0177	0.00990	0.0561	-0.0770
Intrinsic Pathway for Apoptosis	207 - 5366	0.0149	0.00990	0.0663	-0.0559
Intrinsic Pathway for Apoptosis	208 - 5533	0.00880	0.00990	-0.0480	0.0458
Intrinsic Pathway for Apoptosis	208 - 7531	0.00249	0.00990	-0.00393	-0.0538
Intrinsic Pathway for Apoptosis	10000 - 6774	0.00947	0.00990	0.00581	-0.0915
Intrinsic Pathway for Apoptosis	317 - 637	0.0538	0.00990	-0.106	0.126
Intrinsic Pathway for Apoptosis	317 - 54205	0.0102	0.00990	-0.0573	0.0439
Intrinsic Pathway for Apoptosis	317 - 5595	0.0488	0.00990	-0.190	0.0308

Supplementary Table 21. Ten most significant DC edges identified by *dnapath* for the lung cell line Calu3.

pathway	genes	dc score	p-value	nw1	nw2
Intrinsic Pathway for Apoptosis	207 - 208	0.00246	0.1	-0.0110	0.0386
Intrinsic Pathway for Apoptosis	207 - 10018	0.00107	0.1	-0.0110	0.0217
Intrinsic Pathway for Apoptosis	207 - 708	0.00246	0.1	0.0110	-0.0386
Intrinsic Pathway for Apoptosis	207 - 56616	0.000783	0.1	0.0110	-0.0170
Intrinsic Pathway for Apoptosis	207 - 8655	0.00240	0.1	0.0320	-0.0170
Intrinsic Pathway for Apoptosis	207 - 5599	0.00288	0.1	-0.0320	0.0217
Intrinsic Pathway for Apoptosis	207 - 23368	0.00499	0.1	-0.0320	0.0386
Intrinsic Pathway for Apoptosis	207 - 6774	0.00246	0.1	-0.0110	0.0386
Intrinsic Pathway for Apoptosis	207 - 7159	0.00107	0.1	-0.0110	0.0217
Intrinsic Pathway for Apoptosis	207 - 8626	0.00147	0.1	0.0214	-0.0170
