# Supplementary material for: Identification of shared biological features in four different lung cell lines infected with SARS-CoV-2 virus through RNA-seq analysis
Source: Front Genet. 2023 Aug 16;14:1235927. doi: 10.3389/fgene.2023.1235927 (PMC10468990; doi:10.3389/fgene.2023.1235927)
Supplement: Supplementary file 2 [file Image1.PDF]

Supplementary Material

1 DATA PRE-PROCESSING

1.1 Median $\log_2$ Transformed Counts per Gene per Million Mapped Reads (CPM) Distribution

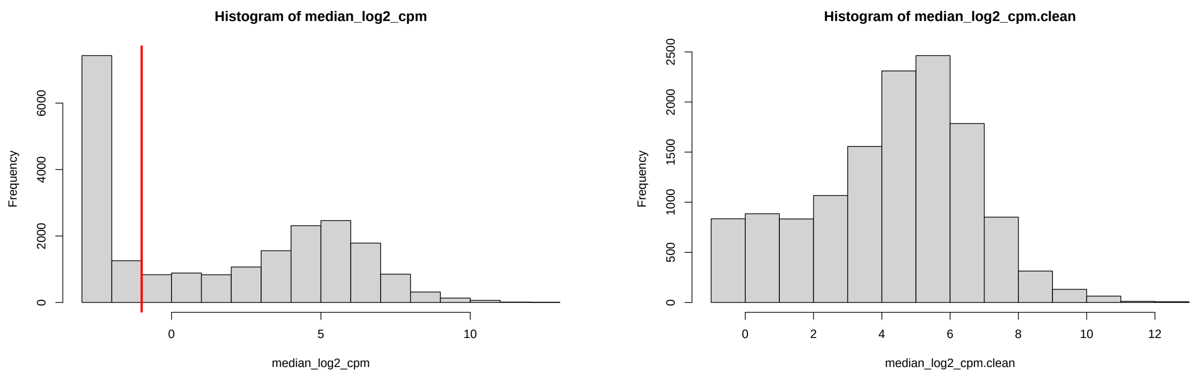

Supplementary Figure 1. Histogram of median $\log_2$(CPM) before (left panel) and after (right panel) removing genes with a median $\log_2$(CPM) below -1. The red line in the left sub-figure represents the cut-off value of median $\log_2$(CPM) (-1).

1.2 Multidimensional scaling (MDS) Plot

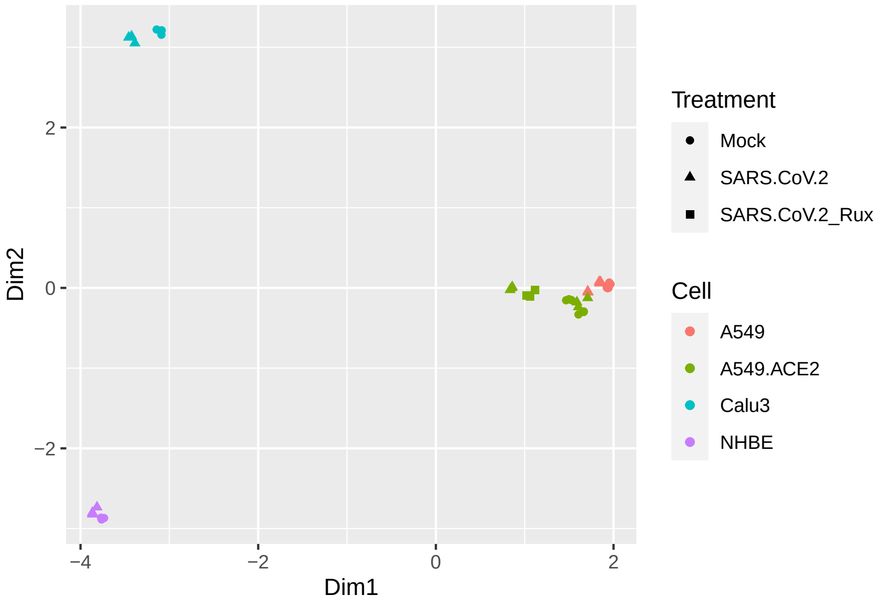

Supplementary Figure 2. MDS plot measuring the distances between two groups, with each color representing distinct cell lines and each shape representing different treatment groups.

1.3 Heatmap Plot

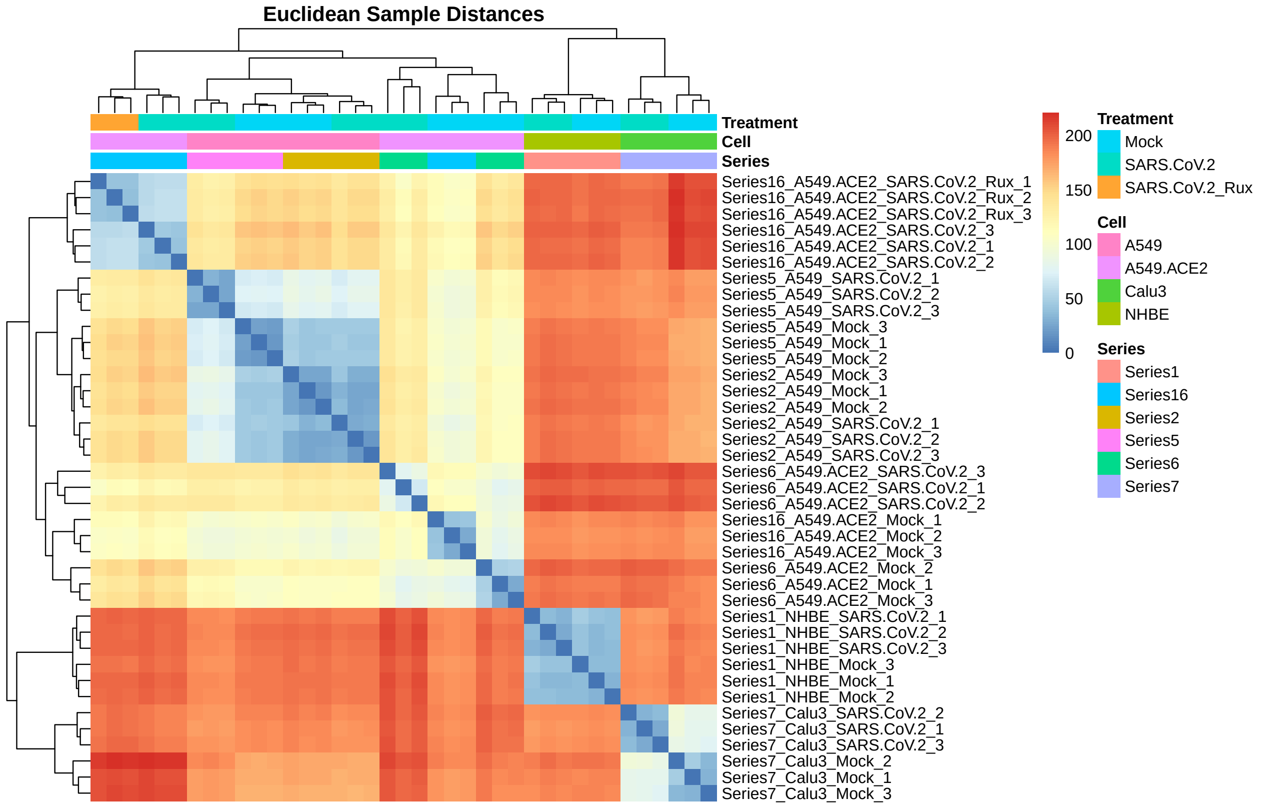

Supplementary Figure 3. Heatmap plot displaying the distances between two groups, where the three treatment groups are differentiated by color and the four lung cell lines are also represented by distinct colors. Additionally, series are indicated using different colors to signify each experiment number.

2 DIFFERENTIAL EXPRESSION ANALYSIS

2.1 Volcano Plots

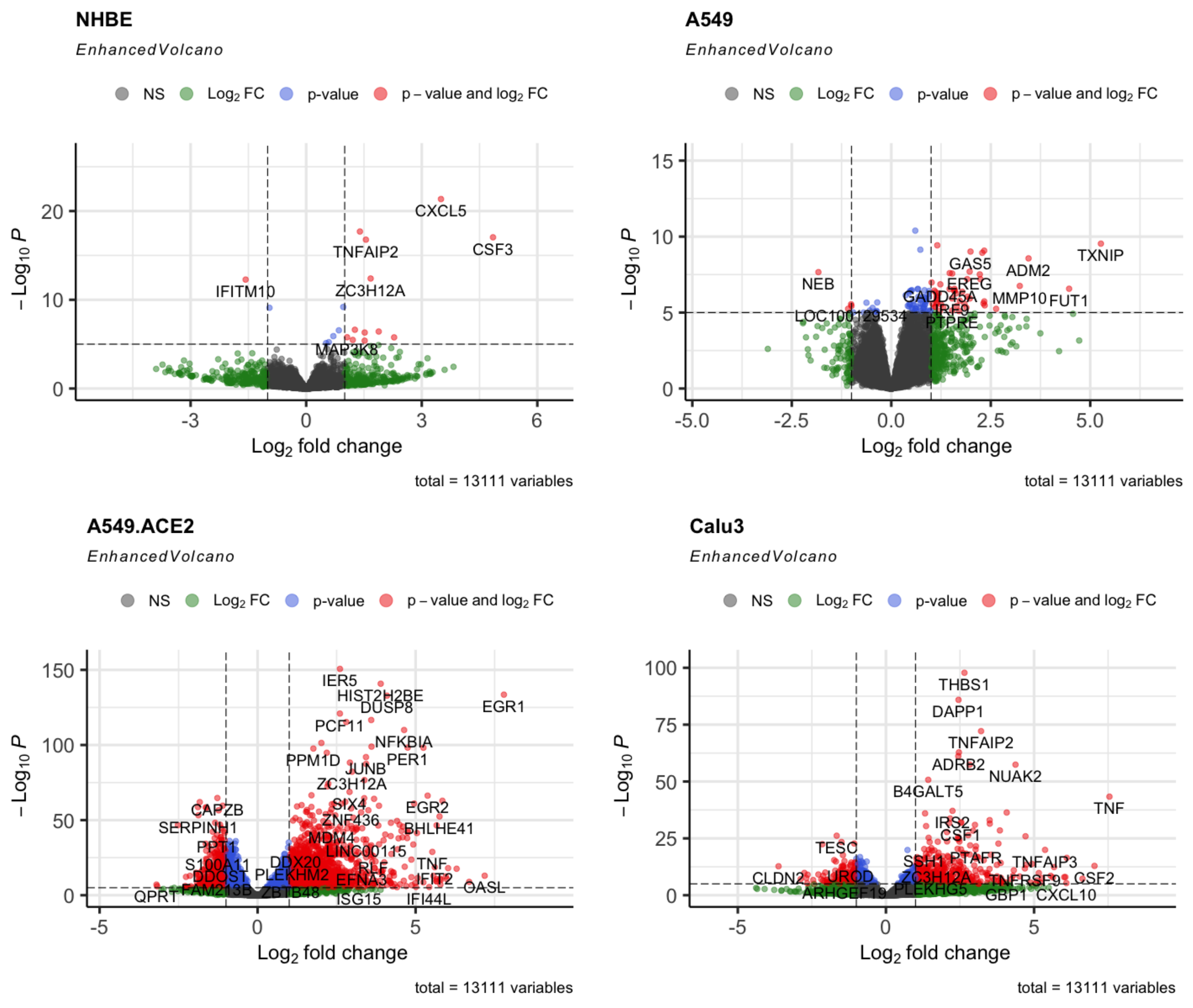

Supplementary Figure 4. Volcano plots for each cell line type

3 PATHWAY ANALYSIS

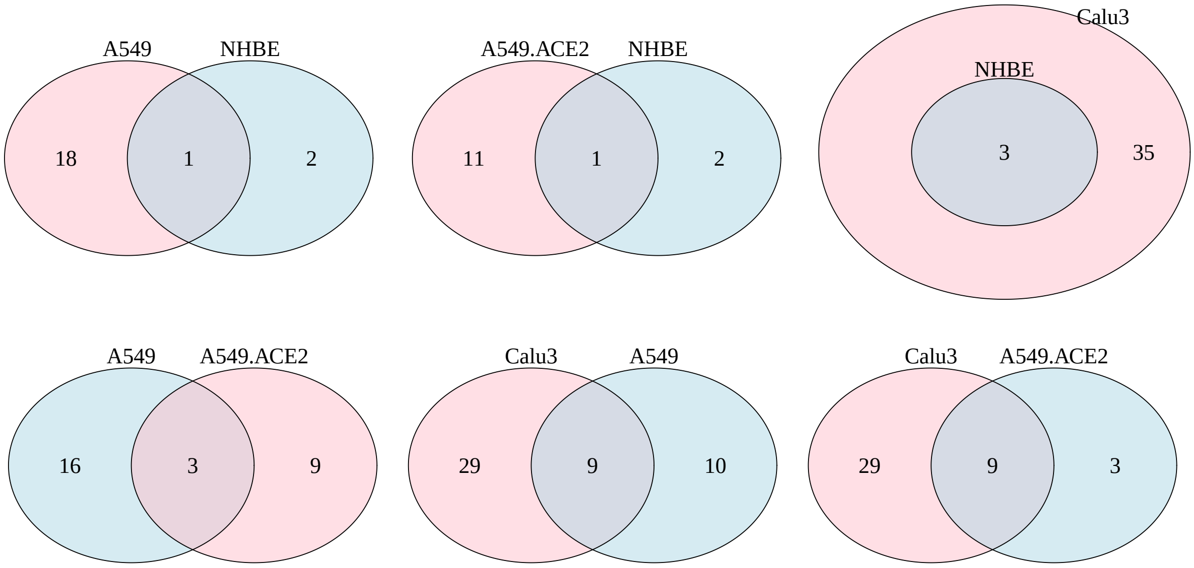

Supplementary Figure 5. Venn diagrams comparing significant signaling pathways among the four lung cell lines: A549 vs NHBE, A549.ACE2 vs NHBE, Calu3 vs NHBE, A549 vs A549.ACE2, A549 vs Calu3, and A549.ACE2 vs Calu3. To be specific, A549 vs NHBE: "Cytokine-cytokine receptor interaction"; A549.ACE2 vs NHBE: "Cytokine-cytokine receptor interaction"; Calu3 vs NHBE: "Cytokine-cytokine receptor interaction", "Chemokine signaling pathway", and "Pertussis". A549 vs Calu3: "Cytokine-cytokine receptor interaction", "Rheumatoid arthritis", "MAPK signaling pathway", "Jak-STAT signaling pathway", "Melanoma", "Pathways in cancer", "HTLV-I infection", "Malaria", and "Osteoclast differentiation"; A549.ACE2 vs Calu3: "Circadian rhythm - mammal", "Huntington's disease", "Parkinson's disease", "Herpes simplex infection", "Cytokine-cytokine receptor interaction", "Amyotrophic lateral sclerosis (ALS)", "NF-kappa B signaling pathway", "Alzheimer's disease", and "Rheumatoid arthritis"
